# Subdermal Laser‐Assisted Scar Subcision (SLASS) Combined With Fractional CO_2_ Laser for Acne Scars: Efficacy Evaluation

**DOI:** 10.1111/jocd.70219

**Published:** 2025-04-29

**Authors:** Adrián Alegre‐Sánchez, Ana Suárez‐Valle, Diego Fernández‐Nieto, Alejandro Lapeña, Elena Macías‐del Toro, Claudia Bernárdez

**Affiliations:** ^1^ Dermatology Clinic AB Derma Madrid Spain

**Keywords:** acne scars, CO_2_ laser, endolaser, endolift, rolling scars, subcision

## Abstract

**Background:**

Rolling atrophic acne scars attach the deeper dermis to the muscular–fascial layer with a fibrous component. Subcision is the most commonly used treatment technique for rolling scars.

**Aims:**

Our objective is to describe the efficacy and safety profile of a new type of subcision: the “subdermal laser‐assisted scar subcision” (SLASS), using a 1470‐nm diode fiber laser in combination with ablative fractional CO_2_ laser.

**Patients/Methods:**

A retrospective study of the combination of subcision with subdermal laser and CO_2_ fractional laser in one session for 42 patients is presented. For the subcision, 400‐μm fibers of a 1470‐nm diode laser were used with energies ranging from 100 to 200 J per 2 cm^2^ area, using 3–4 W of power and pulsed mode with 15–25 ms pulses. After subcision, CO_2_ fractional laser was performed with different settings depending on the deepness of the scars: 40–50 W power peak, 1–1.5 ms dwell time, and 20%–30% density.

**Results:**

Mean reduction of the ECCA grade scale before and after treatment was of 92.6 ± 34.3 points (*p* < 0.05). Considering SCAR‐S scale, ranging from 0 to 5 (0 = clear; 5 = very severe), a mean improvement of 1.67 points was obtained (*p* < 0.05). Significant differences were found in both ECCA scale and SCAR‐S scale before and after treatment. None of the patients had severe adverse effects after the procedure.

**Conclusions:**

SLASS subcision can be considered as a promising technique for rolling acne scars. It provides a thermo‐mechanical subcision effect to the scarred area compared with traditional subcision techniques. CO_2_ laser seems effective and safe.

## 1 Introduction

Acne scars profoundly impact the emotional and psychological well‐being of patients. The persistent nature of these scars can lead to diminished self‐esteem, social anxiety, and even depression [1]. Different treatment tools can be used for their improvement, but up to now there is no treatment that can improve them completely.

Atrophic acne scars are the most common type of acne scars and can be classified as ice‐pick scars, boxcar scars, and rolling scars [2]. Rolling scars are the deepest ones, with a very important fibrous component attaching to the deeper layers of the dermis and the muscular‐fascial layer of the face, making these scars difficult to treat with most energy‐based device treatments or other tools.

Historically, various treatment modalities have been employed to combat acne scars. These include chemical peels, microdermabrasion, microneedling, and laser resurfacing, each offering varying degrees of success and limitations. Chemical peels and microdermabrasion often require multiple sessions and achieve only superficial improvement. Microneedling, while effective in stimulating collagen production, may not sufficiently address deeper scar tissue. Laser resurfacing provides a more direct approach to remodeling scarred skin but can involve significant downtime and risk of pigmentation changes, and normally it does not have a significant impact for rolling scars [3, 4, 5].

For rolling scars, the subcision technique is typically recommended to remove the fibrous component under the scars and elevate the skin surface. In recent years, different subcision techniques have emerged, aiming to either enhance the efficacy of scar treatment with better results or provide safer treatments with less invasiveness [6]. The problems with most of the tools currently used for subcision are that they are difficult to manage, painful, and many times linked to side effects such as bruising, long recovery, or even nerve damage. The exploration of new alternative techniques to better address these difficult scars is needed. Also, on many occasions, patients seek treatments many years after the active acne crop that generated the scars, leading to a situation in which not only atrophic scars are present but also skin laxity and sagging of the face.

In this article our objective is to present an alternative to previous subcision methods: the “subdermal laser‐assisted scar subcision”, using a 1470‐nm diode fiber laser. This method had already been proposed in a small pilot study in Nilforoushzadeh et al. [7]. Here we report a larger series of patients, with newly explained settings of the device and a more complete description of the technique. Also, the combination of this new method with a more traditional treatment for acne scars such as CO_2_ fractional ablative laser is proposed for the first time. We tried to evaluate the safety and efficacy of the combination of SLASS subcision and CO_2_ laser for rolling scars. The use of subdermal laser in rolling acne scars can have a double effect: on one hand, it generates a thermomechanical subcision of the scars and on the other hand, it can also be effective for the treatment of skin laxity, providing a lifting‐like effect of the area.

**FIGURE 1:**
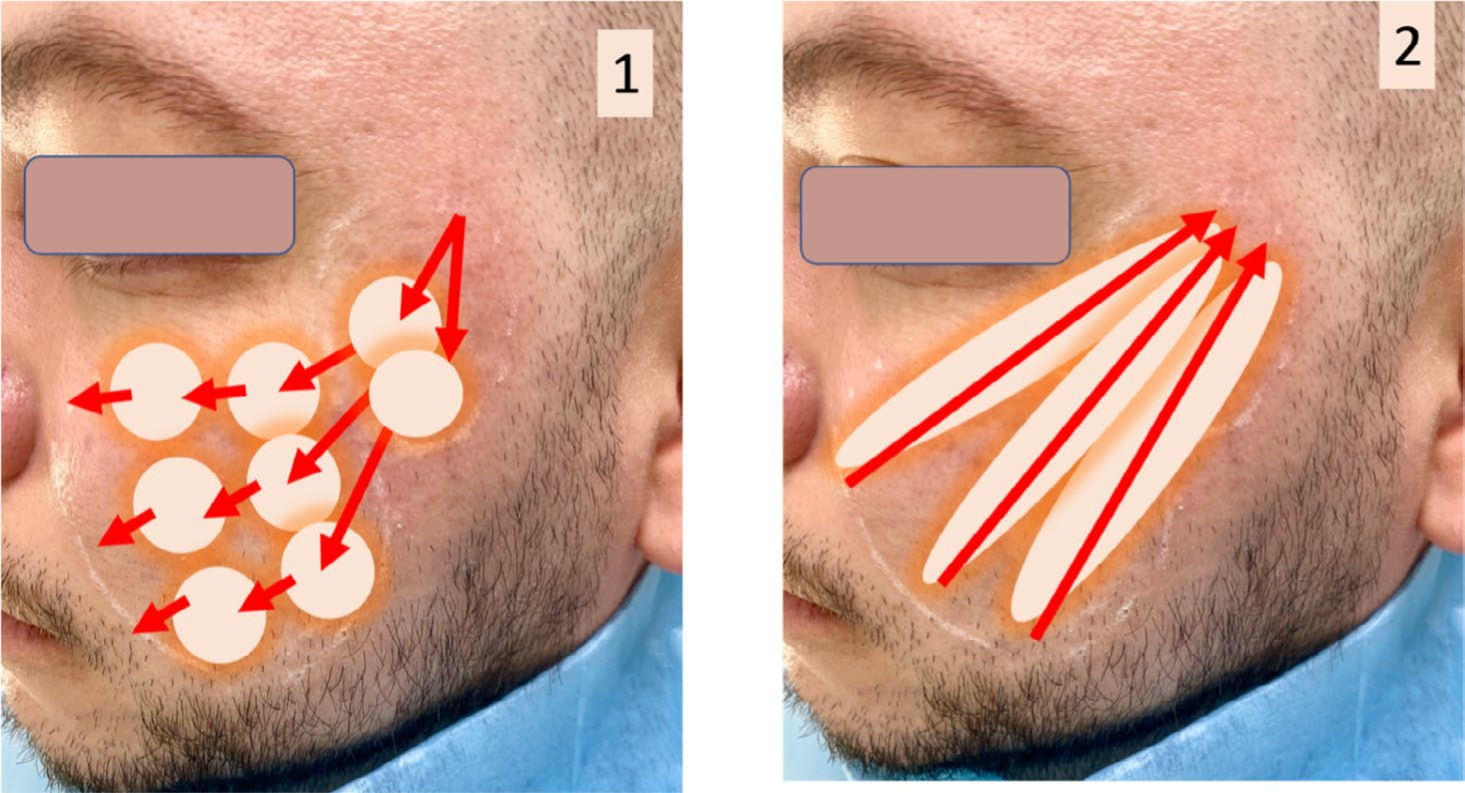
Schematic representation of the treatment technique with subdermal fiber laser: (1) First step with anterograde movements with 15 ms on/65 ms off pulsed mode of the laser to ablate the fibrous component. (2) Second step in a retrograde movement for tightening effect with 25 ms on/75 ms off mode.

**FIGURE 2:**
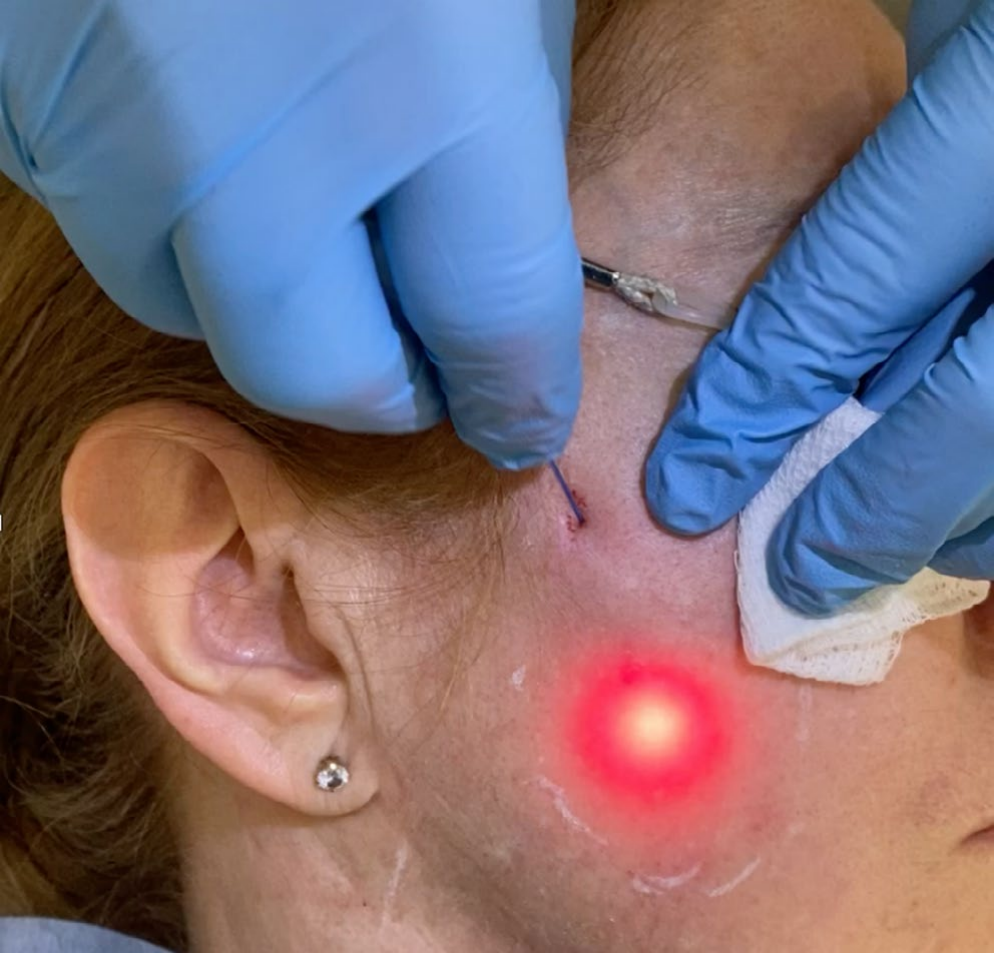
Image of the 400‐μm fiber laser emitting 1470‐nm in the subdermal layer.

**FIGURE 3:**
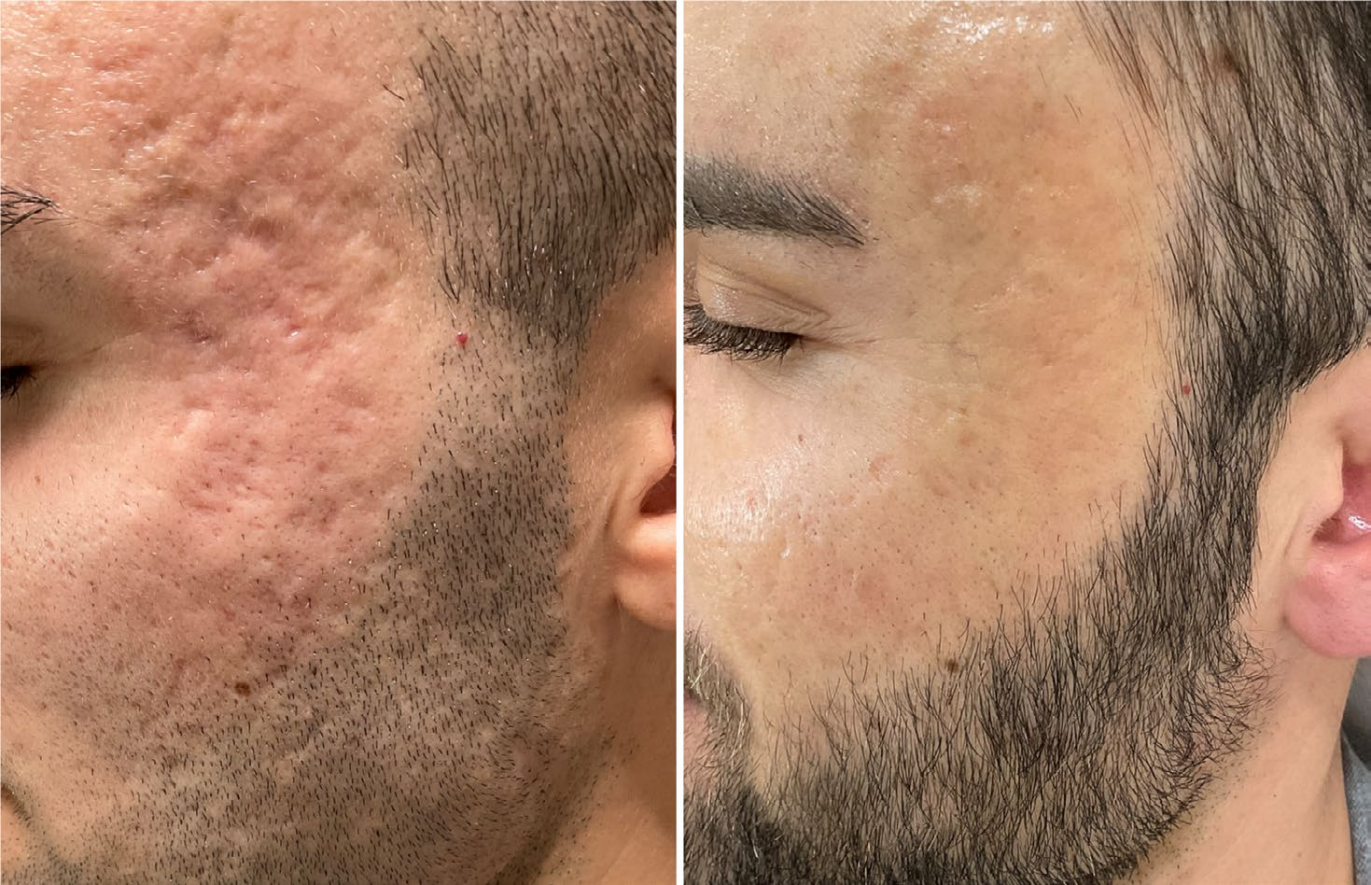
Before (left image) and after (right image) treatment with the combination of SLASS subcsion and fractional CO_2_ laser. After image is 12 weeks after one session of treatment.

**FIGURE 4:**
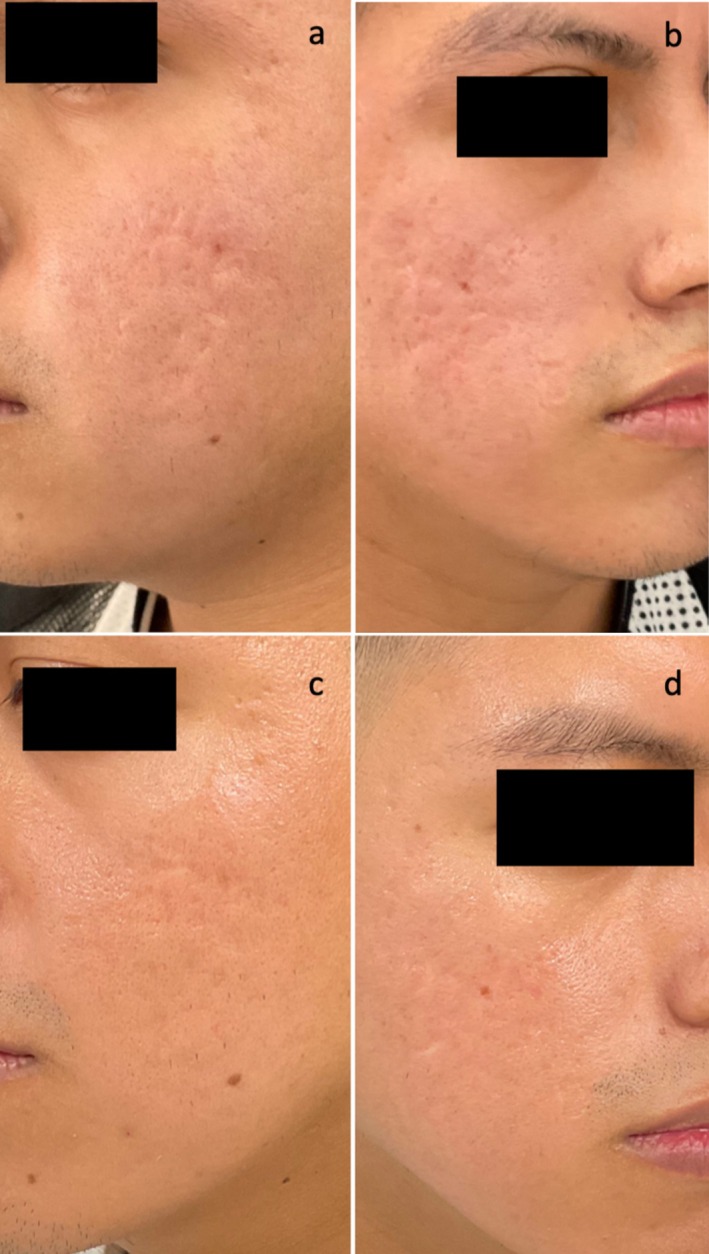
Before (a, b) and after (c, d) treatment with the combination of SLASS subcsion and fractional CO_2_ laser. After image is 12 weeks after one session of treatment.

## 2 Material and Methods

The study was performed in the Dermatological and Aesthetic Clinic ABderma in Madrid, Spain. All the data available and clinical pictures from patients treated with the combination of subcutaneous 1470 nm fiber (Lasemar 1500 from Eufoton s.r.l, Italy) laser and fractional CO_2_ (Smartxide Touch laser from DEKA laseres, Italy) laser from 2022 to 2024 were collected. Patients that had received any other treatment apart from this combination (i.e., fillers, biostimulators, other lasers) were excluded from the study. Patients treated with this combination but lacking pictures of before and after treatment or any other treatment settings information were excluded. Although some of the patients had received more than one session of this combination or had received different treatments afterward, only the pictures from before and after the first combined session SLASS + fractional CO_2_ laser were analyzed.

In total, 42 patients were included in the study: 29 female patients and 13 male patients. The mean age of the participants was 28 years. All the patients were contacted and signed an informed consent prior to their inclusion in the study.

Treatment was performed by first applying topical anesthesia with a mixture of lidocaine at 7%, prilocaine at 7%, and tetracaine hydrochloride at 6% for 15–20 min, followed by infiltration of 2–3 mL of local anesthesia using a cannula with lidocaine at 2%. Regarding the procedure, the first step in all patients was always the subcutaneous laser‐assisted subcision with a 1470 nm fiber laser. Areas of up to 2 cm^2^ were first sterilized and outlined by a surgical marker with the patients sitting and with an indirect light from above that enhanced the shadow of the scars.

The treatment was performed with 400‐μm fibers, instead of the typical 200‐ or 300‐μm fibers used for face tightening. This fiber is more easily passed through the fibrous septums underneath acne scars without bending or changing the treatment plane. The fiber was injected without a previous needle‐made entry point, just by heating rapidly the epidermis and pushing it through the skin. A superficial subdermal plane was maintained for all the treatment. Firstly, fast forward movements against the fibrous tissue were performed (image 1) without firing the laser fiber in all the path, only when a fibrosis was noted, until it was ablated. These forward movements heating and ablating the fibrous septum underneath the scars were repeated until all the areas were noticed as separated and no obstacles to the movement of the fiber were encountered. The following settings were chosen for this first ablative step: 3–4 W, 15 ms on/65 ms off for a total of 100–200 J. Additionally, when treating thicker male skin, if the ablation of fibrosis became complicated, we adjusted the settings of this first step from 15/65 to 25/65 to facilitate the process.

Then, in the second phase of the treatment, the tightening step of the SLASS, a gentle and slow retrograde movement with the fiber, was performed in all the area with 3–4 watts, always with 25 ms on/75 ms off, summing up to a total of 150–200 J more (Figures 1, 2).

After the SLASS, the CO_2_ laser treatment was performed. Smartxide touch2 laser (DEKA, Italy) was used for the CO_2_ treatment with the following settings: power from 40 to 60 W; dwell time 1000–1500 μs; separation space 300–700 μm (equivalent to a density of 30%–20%). More aggressive settings were used for the areas with more atrophic scars and less intensive settings for the surrounding areas. The after‐treatment care was done with topical antibiotic (Fusidic acid once daily for 5 days), sun protection, and recovery cream.

The results were evaluated by two blinded dermatologists using the Global Scale for Acne Scar Severity (SCAR‐S) and the ECCA grading scale (*échelle d'évaluation clinique des cicatrices d'acné*) [8, 9]. Results were also collected from the patients using a subjective scale from 0 (no response) to 10 (total response).

## 3 Results

All the patients included retrospectively had their photographs analyzed by two blinded dermatologists and were contacted via phone or email to answer the subjective scale of improvement. Post‐treatment evaluation pictures were available with an interval from treatment of 16.6 weeks, considering all cases.

Regarding the evaluation by blinded dermatologists, mean ECCA scale grade before treatment obtained a result of 172.02 ± 40.8. ECCA scale grade after treatment was 79.40 ± 24.35, with a mean reduction of ECCA grade of 92.6 ± 34.3 points (*p* < 0.05). Considering SCAR‐S scale, ranging from 0 to 5 (0 = clear; 5 = very severe), a mean improvement of 1.67 points was obtained (*p* < 0.05), from a mean evaluation of 4.02 ± 0.74 before treatment to 2.36 ± 0.87 after treatment. Significant differences were found in both ECCA scale and SCAR‐S scale before and after treatment (Figures 3, 4).

Mean subjective improvement perceived by patients was 7.02 ± 1.3 on a scale from 1 to 10. This can be considered a very good improvement. No differences were found in terms of efficacy or safety based on sex, age, or skin phototype among the patients.

None of the patients had severe adverse effects after the procedure. The downtime period included 5–7 days of swelling, edema, and crusts mainly due to CO_2_ laser treatment. Some of the patients reported mild paresthesia in the area for some weeks after SLASS with subdermal laser. None of our patients experienced complications related to motor nerves.

## 4 Discussion

Treatment of acne scars is very challenging, and it needs to master different techniques from surgical techniques to energy‐based devices or injectables. New treatments are continuously being tested alone or in combination to achieve better results. In this study, we propose the combination of a more common technique for the treatment of acne scars (CO_2_ ablative laser) with a novel technique to perform the subcision of the scars (SLASS or subdermal laser‐assisted scar subcision).

Subcision itself is a technique well‐described in the literature from 1995 used to treat atrophic scars [10]. Its main objective is to remove the fibrous septum under the scar, in a subcutaneous plane. This fibrous component attaches the scar to deeper muscular‐fascial planes and makes it responsible for the “rolling” scar that we prefer to call attached scars. Subcision has been demonstrated to be effective and safe for the treatment of this type of scars using different techniques and devices. The Nokor needle is a classical tool used for subcision, but also different types of “liberators” or blunt cannulas have demonstrated to be effective for the treatment [6]. One of the main problems of previous subcision techniques is that the treatment itself was only a mechanical procedure, cutting or moving the fibrous component of the scar but without having any impact on the stimulus of neo‐collagen production in the deeper layers of the dermis. Also, more aggressive tools such as liberators are very often related to pain, bruising, or even nerve or ligament damage after procedures. Additionally, this type of tool requires a long training period for the surgeon performing the treatment in order to achieve better results, and the results are not very reproducible.

In this article we provide a new type of subcisson that we call “subdermal laser‐assisted scar subcision”, to emphasize the aspect that a subdermal laser fiber is used as the tool to perform subcision. Only one previous study had been published using a fiber subcutaneous laser to treat rolling acne scars. In the pilot study of Nilforoushzadeh et al. the same device was used (LaseMar 1500, Eufoton, Italy) but with thinner fibers and different parameters: 50 ms on and 75 ms off. We propose a more adapted parameters of the laser using different settings for an ablative step of the procedure (15 ms on, 65 ms off), given that in this step the objective is to remove all the fibrous components and adding a second step with the fiber laser with a lifting effect (25 ms, 75 ms off in a retrograde movement). Also, the use of thicker fiber of up to 400 μm for acne scar is more adequate considering the stiffness of the skin in the areas of scarring, and it has allowed us to perform a more effective subcision of the scars. With the SLASS technique using thicker fibers, it can be considered that the subcision is due to both thermal and mechanical effects due to the action of the heat delivered by the laser beam and the movements of the fiber under the scars. Thus, we propose the term of thermomechanical subcision as the mechanism of action of the SLASS technique. We prefer the term of subdermal instead of subcutaneous laser to enhance the fact that we are treating the subcutaneous plane but just under the dermal layer because in this layer is where most of the fibrous septums of the scars are attached.

Another advantage of SLASS subcision is that it only needs one or a few entry points per side of treatment, compared to the subcision with previous tools such as Nokor needle that need to perforate the skin in smaller areas. Also, the thermal effect of the laser is going to produce not only the subcision of the scars but also the remodeling of the collagen in the deeper dermis and stimulation of new collagen production. Compared to other mechanical tools for subcision such as liberators, with the laser fibers, minimal or no bruising is produced due to the coagulative effect of the diode laser.

The addition of the treatment with the fractional CO_2_ laser after the SLASS subcision allows stimulating the production of neocollagenesis during the recovery period of the subcision, allowing for a faster and stronger response to the treatment in just one session. Up to now, this is the first study demonstrating the safety and efficacy of the combination of both subcision with a laser fiber underneath the dermal layer and fractional ablative laser for the upper epidermis and dermal layers.

One limitation of the subdermal‐laser‐assisted scar subcision might be its cost‐effectiveness. The laser fibers used in our study do have a cost associated with each patient; however, they can be re‐sterilized and utilized up to 10 times, which mitigates the expense over multiple uses. While we acknowledge that the initial cost is higher than traditional mechanical subcision—approximately 20 times greater in material costs—we believe that the effectiveness of the endolaser justifies this investment. All patients in our study accepted this increased cost without issue.

It is acknowledged that this is an exploratory and retrospective initial report of this combined technique utilizing subcutaneous endolaser and CO_2_ laser in the same session. Future studies that comparatively analyze the differences in efficacy and safety between subcision assisted by subcutaneous laser and traditional subcision would be very interesting.

In all, SLASS subcision with CO_2_ ablative fractional laser seems to be a promising treatment combination for acne scars with a rolling component. However, more controlled or split‐face treatment comparing SLASS subcision with other techniques should be performed in order to better address its efficacy and safety.

## 5 Conclusion

A new combination of treatments with energy‐based devices is proposed for rolling acne scars. The term “subsermal laser‐asissted scar subcsision” is proposed as a global term referring to the use of fiber lasers under the dermal layer to ablate the fibrous component of rolling scars and generate a remodeling of the deep dermis in acne scars.

## Author Contributions

All the authors included have contributed to the elaboration of the study, either with the treatment of patients included, the blinded evaluation, or the redaction of the main manuscript.

## Ethics Statement

The authors confirm that the ethical policies of the journal, as noted on the journal's author guidelines page, have been adhered to. No ethical approval was required as this is a retrospective study. All the patients included signed informed consent for both the treatment and the use of their data and/or images for the present study.

## Conflicts of Interest

The authors declare no conflicts of interest.

## Data Availability

The data that support the findings of this study are available on request from the corresponding author. The data are not publicly available due to privacy or ethical restrictions.
